# Suicidal Ideation Across the Lifespan Among Commercially Insured Outpatients: Retrospective Cohort Study

**DOI:** 10.2196/101421

**Published:** 2026-08-19

**Authors:** Dena M Bravata, James M Hudson, Severin Kibby, Neha P Chaudhary, Taft Parsons III, Nicole M Benson

**Affiliations:** 1 Center for Primary Care and Outcomes Research, Stanford University, Palo Alto, CA, United States; 2 TherapyMatch (dba Headway), New York, NY, United States; 3 Harvard Medical School, Massachusetts General Hospital, Boston, MA, United States; 4 CVS/Aetna, Detroit, MI, United States; 5 Harvard Medical School, McLean Hospital, 115 Mill St, Belmont, MA, 02478, United States, 1 617-855-2000

**Keywords:** suicidal ideation, mental health, outpatient care, telehealth, behavioral health, adolescents, commercially insured

## Abstract

**Background:**

Suicide remains a leading cause of death in the United States and is on the rise. Limited evidence describes the current burden of suicidal ideation (SI) among commercially insured outpatients, a population that represents a large and rapidly expanding segment of those seeking care.

**Objective:**

The objective of this study was to characterize the prevalence of SI among a national cohort of commercially insured individuals presenting for outpatient mental health services, and to examine differences across key demographic and social factors.

**Methods:**

This was a retrospective evaluation of patients, aged 6 to 64 years, presenting for their first visit with an outpatient mental health -clinician between January 1, 2024, and October 31, 2025. The prevalence of SI (defined as a nonzero response on question 9 of the Patient Health Questionnaire-9) was compared by age, sex, and geography using regression analysis.

**Results:**

Among 189,225 commercially insured individuals included in the analysis, mean age was 33.60 (SD 11.33) years. Among those reporting sex, 68% (34,168/50,378) identified as female, 29% (14,491/50,378) as male, and 3% (1719/50,378) as nonbinary. Most had a diagnosis of anxiety (56,176/189,225, 29.7%) or depression (50,075/189,225, 26.5%). In total, 18.6% (35,215/189,225) of individuals indicated some level of SI. Across age groups, SI was highest among adolescents (age 13‐17 years) with 32.8% (2107/6426) reporting any suicidality, and 4.8% (306/6426) reporting SI nearly every day (*P*<.001). The population with the greatest SI comprised individuals identifying as nonbinary (644/1719, 37.5%; *P*<.001). SI increased as a function of patients’ social vulnerability (*P*<.001) and varied by geography.

**Conclusions:**

Nearly 1 in 5 individuals endorsed some degree of SI, with one third of adolescents reporting SI, proportions that are notably higher than estimates from the general US population. These findings underscore the need for comprehensive screening for SI and the expansion of clinical support for suicidal populations.

## Introduction

Suicide remains a leading cause of death in the United States, with rates of suicide and suicidal behaviors rising over recent decades despite national prevention initiatives. Suicide is the 10th leading cause of death in the United States [1], accounting for approximately 49,000 deaths annually and more than 800,000 deaths worldwide [2,3]. Suicidal ideation (SI) is more prevalent than suicide attempts or deaths and represents a critical precursor in the pathway to self-harm. In national surveillance data, 5.3% of US adults reported serious suicidal thoughts in the preceding 12 months, with disproportionately higher rates among adults aged 18 to 24 years, those with lower household income, and individuals identifying as bisexual [3]. More than 2 in 5 US adults report knowing someone who died by suicide, an exposure associated with elevated suicide risk [3].

Rates of SI are substantially higher among individuals seeking outpatient mental health care. In a recent study, 19% of outpatients endorsed suicidal thoughts (including 11% with current ideation), and among adults with major depressive episodes, a diagnostic group common in outpatient settings, 26% to 33% reported SI in the past year, with increases observed over the last decade [4]. The rate of SI among children and adolescents in the United States varies by age group with approximately 15% of preadolescents (under age 13) and 13% to 22% of adolescents (aged 13 to 17) reporting SI annually [5]. The burden of SI is further compounded by social determinants of health, including experiences of trauma, minority status, and socioeconomic disadvantage, which are strongly associated with increased suicide risk and highlight the need for comprehensive risk assessment in clinical practice. Despite the high prevalence of SI and attempts, a significant proportion of at-risk individuals do not receive timely or adequate mental health services, underscoring persistent gaps in care and the importance of both clinical and public health strategies to address suicide risk in outpatient settings.

Despite this growing body of literature, limited evidence describes the burden of SI among commercially insured outpatients, a population that represents a large and rapidly expanding segment of mental health service users. The objective of this study was to characterize the prevalence of SI among a large, national cohort of commercially insured individuals presenting for outpatient mental health services and to examine differences across key demographic and social factors. Understanding patterns of SI in this population may help inform risk stratification efforts and guide the development of timely, scalable interventions to support patients at elevated risk for suicide.

## Methods

### Study Population

This was a retrospective cohort evaluation of patients presenting for their first visit with an outpatient mental health clinician between January 1, 2024, and October 31, 2025, in the Headway mental health clinician network. Headway is the largest behavioral health network in the country, comprising 60,000 clinicians who have more than 1 million patient visits per month [6]. We excluded patients without commercial insurance (ie, Medicaid or Medicare coverage or self-pay).

### Data Sources

We collected all patient, -clinician, and visit information from the Headway digital platform. Patient information included patient age, sex, home zip code, clinical condition, and insurance type. Clinician type was classified as psychotherapist or psychiatric clinician.

To assess SI at presentation, we extracted responses on the Patient Health Questionnaire-9 (PHQ-9) at participants’ first visit [7]. Notably, we did not require the use of the PHQ-9 by clinicians; rather, we reported on the outcomes collected by clinicians and provided via the Headway platform. The ninth question of the PHQ-9 has been routinely used to screen for suicidal thoughts [8-10] and asks, “Over the last two weeks, how often have you been bothered by thoughts you would be better off dead or of hurting yourself in some way?” Responses are scored 0 for not at all, 1 for several days, 2 for more than half the days, and 3 for nearly every day [7].

We assigned a Social Vulnerability Index (SVI) score to each participant based on their home zip code according to the Centers for Disease Control and Prevention (CDC) [11]. The SVI is presented as a percentile from 0 (least vulnerable) to 1 (most vulnerable) that allows geographies to be directly compared based on 16 measures across 4 categories per geography: socioeconomic status, household characteristics, racial and ethnic minority status, and housing type and available transportation [11].

### Analysis

We compared the prevalence of SI (as defined by a nonzero response on question 9 of the PHQ-9) among patients presenting for initial outpatient care by age, sex, geography, and clinician type using univariate analyses. We used regression analysis to evaluate which of these characteristics were most associated with SI.

For geographic analyses of SI in the United States, we sought to evaluate the burden of SI in the population compared with the availability of mental health clinicians in each state. Specifically, we computed an SI Care Gap Index, which we calculated as the rate of SI in each state (from our analyses) divided by the number of mental health clinicians per population as reported by the Kaiser Family Foundation (KFF) [12]. An SI Care Gap Index of 1.0 indicates a state with average SI prevalence and average mental health workforce; values>1.0 indicate a dual burden of elevated SI prevalence and mental health workforce shortage; values approaching 2.0 indicate the most acute dual burden.

### Ethical Considerations

This study was determined to be exempt by the WIRB-Copernicus Group Institutional Review Board (Headway.001; July 17, 2025). We followed the cross-sectional STROBE (Strengthening the Reporting of Observational Studies in Epidemiology; Checklist 1) [13] reporting guidelines.

## Results

### Participants

189,225 commercially insured individuals were included in this study. The mean age of the population of 185,913 for whom age was available was 33.60 (SD 11.33) years. Among those reporting sex, 68% (34,168/50,378) identified as female, 29% (14,491/50,378) as male, and 3% (1719/50,378) as nonbinary. Most patients presenting for outpatient mental health care had a diagnosis of anxiety (56,176/189,225, 29.7%), depression (50,075/189,225, 26.5%), or adjustment disorders (40,102/189,225, 21.2%). Nearly three-quarters of patient visits (138,259/189,225, 73%) were with a psychotherapist (50,966/189,225, 27% were with a psychiatric prescriber).

### SI

The mean PHQ-9 score among the total presenting population was 9.69 (SD 6.25; Table 1). Of these, 81.4% (154,010/189,225) reported no SI (mean PHQ-9 score 8.24, SD 5.46), while 18.6% (35,215/189,225) indicated some level of SI (mean PHQ-9 score 16.03, SD 5.50). Specifically, 13.5% (25,499/189,225) indicated SI for several days, 3.1% (5819/189,225) for more than half the days, and 2.1% (3897/189,225) nearly every day.

**Figure 1.**
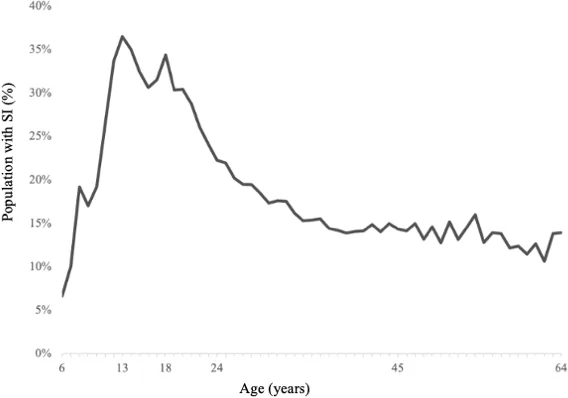
Suicidal ideation (SI) by age. This figure plots age on the x-axis against the percentage of patients with SI on the y-axis and demonstrates a peak among adolescents and young adults.

**Figure 2.**
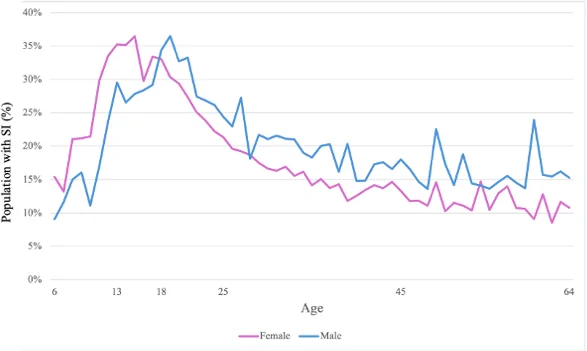
Suicidal ideation (SI) by sex. This figure plots age on the x-axis against the percentage of patients with SI on the y-axis with female individuals in purple and male individuals in blue. It demonstrates that female individuals younger than 18 years have higher rates of SI, but after that age, male individuals consistently have higher rates of SI.

Across all age groups, SI was highest among teenagers (aged 13‐17 years) with 32.8% (2107/6426) reporting any suicidality and, importantly, 4.8% (306/6426) reporting SI nearly every day (*P*<.001; Table 1 and Figure 1). The population with the greatest SI comprised individuals identifying as nonbinary (644/1719, 37.5% vs 3129/14,491, 21.6% for male individuals vs 6,429/34,168, 18.8% for female individuals *P*<.001; Table 1). Notably, SI was higher among female individuals younger than 18 years but then consistently higher for male individuals older than that (Figure 2). Among patients presenting with SI, depression was the most common disorder (14,977/35,215, 42.5%), with anxiety (7411/35,215, 21%) and adjustment disorder less common (4670/35,215, 13.3%; Multimedia Appendix 1). The underlying mental health condition among suicidal patients varied by age: 11.8% (80/677) of children (aged 6‐12 years) with SI had attention-deficit/hyperactivity disorder (Multimedia Appendix 1). In contrast, posttraumatic stress disorder and stress-related disorders were the primary conditions among 9.1% (04/4453) of adults aged 45 to 64 years.

**Table 1.** Suicidal ideation by patient characteristic.

Characteristic	Total PHQ-9^a^ score, mean (SD)	Response to question 9 of the PHQ-9^b^, n (%)			
		0	1	2	3
Overall population (n=189,225), n (%)	9.69 (6.25)	154,010 (81.4)	25,499 (13.5)	5819 (3.1)	3897 (2.1)
Sex, n (%)					
Female (n=34,168)	10.18 (6.27)	27,739 (81.2)	4735 (13.9)	1054 (3.1)	640 (1.9)
Male (n=14,491)	9.79 (6.25)	11,362 (78.4)	2252 (15.5)	523 (3.6)	354 (2.4)
Nonbinary (n=1719)	12.49 (6.05)	1075 (62.5)	433 (25.2)	131 (7.6)	80 (4.7)
Age (y), n (%)					
6‐12 (n=2991)	6.87 (5.66)	2314 (77.4)	440 (14.7)	133 (4.4)	104 (3.5)
13‐17 (n=6426)	10.16 (6.37)	4319 (67.2)	1368 (21.3)	433 (6.7)	306 (4.8)
18‐24 (n=30,559)	11.19 (6.31)	22,378 (73.2)	5573 (18.2)	1523 (5)	1085 (3.6)
25‐44 (n=113,584)	9.50 (6.16)	94,415 (83.1)	14,356 (12.6)	2949 (2.6)	1864 (1.6)
45‐64 (n=32,353)	9.09 (6.26)	27,900 (86.2)	3311 (10.2)	679 (2.1)	463 (1.4)
Social Vulnerability Index quartile, n (%)					
Low (n=8060)	9.13 (6.13)	6694 (83.1)	1001 (12.4)	213 (2.6)	152 (1.9)
Low-mid (n=41,553)	9.24 (6.16)	34,382 (82.7)	5258 (12.7)	1159 (2.8)	754 (1.8)
Mid-high (n=59,431)	9.62 (6.21)	48,585 (81.8)	7868 (13.2)	1774 (3)	1204 (2)
High (n=72,311)	10.05 (6.33)	58,021 (80.2)	10,296 (14.2)	2390 (3.3)	1604 (2.2)
Clinician type, n (%)					
Psychotherapist (n=138,259)	9.16 (6.10)	113,937 (82.4)	18,017 (13)	3781 (2.7)	2524 (1.8)
Psychiatric clinician (n=50,966)	11.11 (6.44)	40,073 (78.6)	7482 (14.7)	2038 (4)	1373 (2.7)
PHQ-9 score, mean (SD)	9.69 (6.25)	8.24 (5.46)	14.60 (5.09)	18.38 (4.43)	21.88 (4.27)

a PHQ-9: Patient Health Questionnaire-9.

b Responses to question 9 of the Patient Health Questionnaire-9 are scored 0 (not at all), 1 (several days), 2 (more than half the days), and 3 (nearly every day).

SI increased as a function of patients’ social vulnerability (*P*<.001; Table 1 and Multimedia Appendix 2). With increasing social vulnerability, patients had more SI and were more likely to report more frequent, including nearly daily SI (Multimedia Appendix 2). Analysis of the subcategories of social vulnerability demonstrated that SI was statistically significantly (*P*<.001 for all associations) associated with increased socioeconomic vulnerability (eg, poverty, unemployment, housing cost burden, and lower educational attainment) and living in at-risk households (eg, those with low English-language proficiency, single parents, and civilians with disabilities).

**Figure 3.**
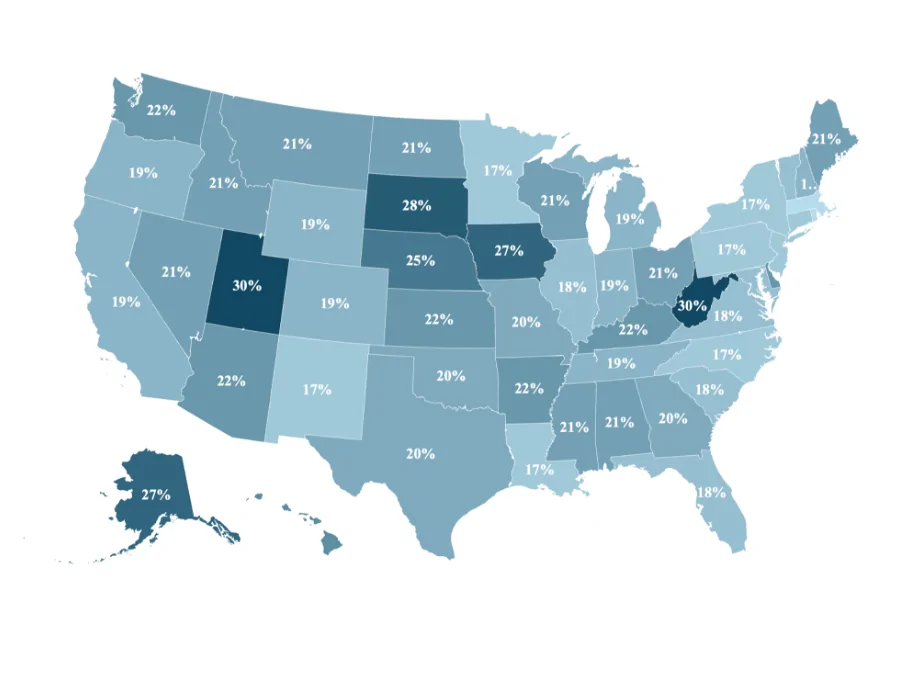
Suicidal ideation (SI) by state. This map of the United States presents the percentage of patients with SI by state, with the District of Columbia and Rhode Island having the lowest rates of SI and West Virginia and Utah having the highest. The colors of each state range from light blue to dark blue along a gradient corresponding the range of SI from 14% to 30%.

**Figure 4.**
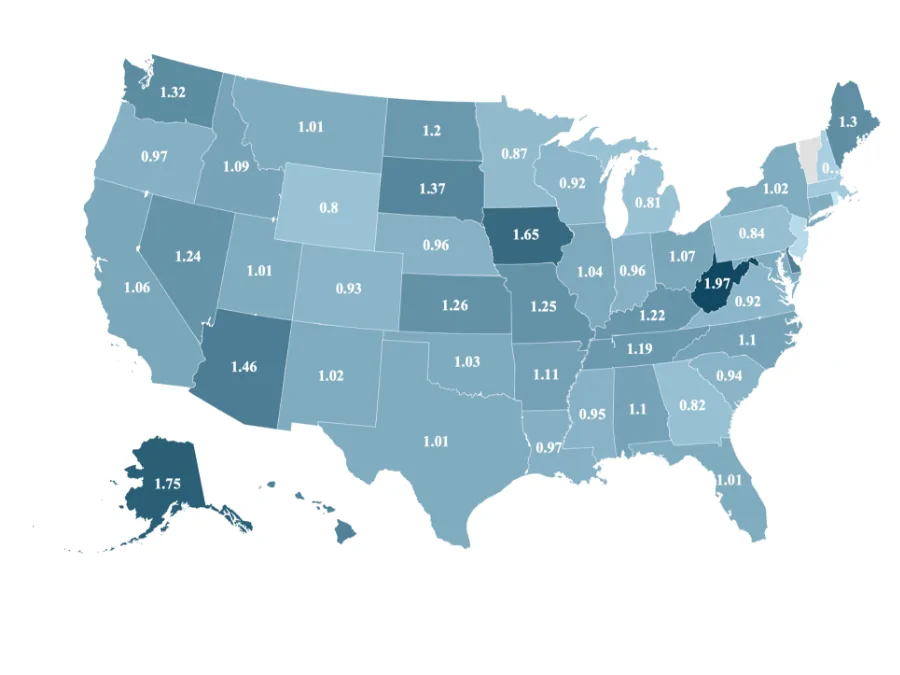
Suicidal ideation (SI) Care Gap Index by state. This map of the United States presents the Care Gap Index by state. It demonstrates that West Virginia and Alaska have the greatest SI care gaps. The colors of each state range from light blue to dark blue along a gradient corresponding the range of the SI Care Gap Index from 0.46 to 1.97.

SI varied by geography with the District of Columbia and Rhode Island having the lowest rates of SI (163/1153, 14.1% and 29/191, 15.2%, respectively) and West Virginia and Utah having the highest (38/125, 30.4% and 274/924, 29.7%, respectively; Figure 3). When comparing the states with high rates of SI to the KFF Mental Health Care Health Professional Shortage Areas, we found that West Virginia and Alaska had the greatest SI care gap (Figure 4). Patients who sought care from a psychiatric prescriber were more likely to have SI than patients seeking care from a psychotherapist (10,893/50,966, 21.4% vs 24,322/138,259, 17.6%; *P*<.001; Table 1).

## Discussion

### Principal Results

The present findings demonstrate that SI is not a marginal phenomenon among commercially insured outpatients but a common and clinically urgent condition, affecting nearly 1 in 5 individuals presenting for mental health care. This prevalence, observed within a population that is insured, engaged in care, and actively seeking treatment, underscores a critical reality: access to mental health services alone is insufficient to mitigate suicide risk. Instead, these data highlight missed opportunities for earlier detection, more precise risk stratification, and targeted intervention across outpatient settings. Moreover, some areas such as West Virginia and Alaska have the dual burden of elevated prevalence of suicidality coupled with shortages of mental health clinicians. These geographies urgently need increased access, including via telehealth, to effective, convenient, and affordable mental health services.

This study has 3 key findings. First, the results presented challenge the assumption that commercially insured populations represent a lower-risk group for suicidality. Much of the suicide prevention literature and public policy focus on uninsured or publicly insured populations; however, our findings indicate that a substantial burden of SI exists even among individuals with financial access to care. Given that commercial insurance covers most working-age adults and many children in the United States, failure to address suicide risk in this group limits the potential impact of national prevention strategies.

Second, the exceptionally high prevalence of SI among children and adolescents, particularly teenagers, represents one of the most concerning findings of this analysis. The rate of any SI among adolescents in this study (2107/6426, 32.8%) far exceeds prior national estimates [3,14,15] and includes a meaningful proportion reporting nearly daily SI, a marker associated with elevated short-term risk of suicide. Importantly, we observed substantial SI among children aged 6 to 12 years, a group for whom routine screening is not universally recommended [16] and for whom validated screening tools are limited. Notably, the PHQ-9 was used by mental health clinicians in this study among younger children despite not having been validated for use in this population. Given the seriousness of this finding, we urge pediatric mental health professionals to screen children presenting for care with validated instruments. Several factors may explain why the rate of SI among children observed in our study was higher than previously reported. Recent studies have demonstrated that SI in children is more common than historically recognized, challenging the longstanding assumption that young children lack the cognitive capacity for suicidal thoughts [5,17]. The COVID-19 pandemic, social isolation, increased screen time, and exposure to psychosocial stressors may be amplifying SI risk in contemporary cohorts [4,15,18]. Among the age-related findings, we hope clinicians, educators, and policymakers will note the striking finding that SI was higher among female individuals younger than 18 years but consistently higher for male individuals aged 18 years and older. Historically, trends have suggested that female individuals have higher rates of SI and attempts, while male individuals have higher rates of death by suicide (largely due to the use of more lethal means) [19]. Recently, the gap in the use of lethal means of suicide between boys and girls has decreased [20], and the sex differences in death by suicide have narrowed. This suggests that suicide prevention efforts in elementary school settings should be sex-specific: for girls, addressing the rising use of lethal methods and the social and digital risk factors such as cyberbullying and social media [20]; and for boys, improving recognition of atypical depression presentations and increasing help-seeking behaviors [21].

Additionally, increased awareness and reduced stigma around mental health may have increased the likelihood of parents and teachers recognizing a child at risk and encouraging them to present for care. Together, these findings suggest that current screening thresholds and age-based recommendations may be insufficient, especially in clinical populations already presenting for mental health care. These findings urgently underscore the need for pediatricians, school-based care clinicians, and others routinely in contact with children to actively screen both boys and girls for suicidal thoughts and make appropriate psychiatric resources readily available to them. For example, school-based mental health awareness programs such as Youth Aware of Mental Health have demonstrated approximately 50% reductions in suicide attempts in large randomized trials [18] and could serve as a model for future interventions.

Third, the observed sex patterns in suicidality further reinforce the need for nuanced prevention approaches. Higher SI rates among girls younger than 18 years, followed by higher rates among adult men, mirror the well-documented divergence between ideation and suicide mortality. This pattern suggests that prevention efforts must be developmentally tailored, emphasizing early identification and support among youth while also addressing lethal means access, substance use, and help-seeking barriers among adult men. The markedly elevated SI prevalence among nonbinary individuals (644/1719, 37.5%) highlights persistent and profound mental health inequities among sex-diverse populations and reinforces the need for affirming, inclusive models of care that extend beyond traditional diagnostic frameworks.

### Implications for Practice and Policy

Several findings from this study point directly toward actionable strategies to reduce suicide risk in the United States. First, the strong and graded association between SI and social vulnerability emphasizes that suicide prevention cannot be disentangled from broader social determinants of health [4,11,14]. Patients living in areas characterized by economic instability, housing insecurity, disability, or limited English proficiency were not only more likely to endorse SI but also more likely to report frequent or near-daily ideation. These patterns argue for integrating social risk screening and navigation services (such as housing support, income assistance, and disability resources) into outpatient mental health care, rather than treating suicidality as an isolated psychiatric symptom. Future studies (especially mixed methods approaches) could be valuable in evaluating whether addressing upstream social needs, such as housing instability, financial stress, or language access to mental health services, reduces the frequency or severity of SI.

Second, the pronounced geographic variation in SI (with rates ranging from approximately 14% to 29% across states), coupled with the SI Care Gap Index, highlights the importance of place-based prevention strategies. States such as Alaska and West Virginia face a dual burden of high SI prevalence and limited access to mental health professionals, conditions that likely contribute to their persistently high suicide mortality rates [22]. These findings support prioritizing telemental health expansion, workforce incentives, and crisis-response infrastructure in high-gap states. At a national level, aligning suicide prevention funding with indicators of unmet need, rather than population size alone, may improve the efficiency and equity of prevention efforts. Moreover, education of primary care clinicians in depression recognition and management represents one of the most scalable and evidence-based suicide prevention strategies with demonstrated reduction in both suicide attempts and deaths [18], and may be particularly relevant in states with high SI care gap indices.

Third, the higher prevalence of SI among patients seeking care from psychiatric prescribers (10,893/50,966, 21.4% vs 24,322/138,259, 17.6% for psychotherapists) underscores the need for universal suicide risk assessment across all outpatient entry points, regardless of clinician type. Whether driven by self-selection, referral patterns [23], or illness severity, this finding suggests that patients at elevated risk may cluster within medication-focused care pathways. Embedding standardized suicide screening, safety planning, and rapid escalation protocols within both psychotherapy and medication management visits could help ensure that risk is identified consistently and addressed proactively.

### Comparisons With Prior Work

Our finding that nearly 1 in 5 outpatients endorsed any SI is consistent with prior reports of approximately 19% suicidal-thought prevalence among outpatient populations [4], but extends that literature by demonstrating that this burden persists in a commercially insured, actively engaged population. The 32.8% (2107/6426) any-SI rate among adolescents in our study substantially exceeds national survey estimates of 13% to 22% for this age group [5,15], likely reflecting both the help-seeking nature of the sample and a contemporary increase in adolescent distress documented in recent national datasets [4,17]. The graded association with social vulnerability complements prior facility-level evidence and underscores the need for upstream interventions.

### Limitations

This study has 3 key limitations. First, the population consisted exclusively of commercially insured individuals, which may limit the generalizability of the findings to patients with Medicaid, Medicare, or no insurance. Second, all SI data were derived from a single PHQ-9 item, which, although widely used and clinically informative, may underestimate or overestimate risk compared with structured interviews. Although item 9 on the PHQ-9 has been shown to be a clinically meaningful, predictive marker of subsequent suicide attempts and suicide death [8-10], it does not distinguish between passive death ideation (“thoughts you would be better off dead”) and active self-injurious ideation (“hurting yourself in some way”). Therefore, we recommend that clinicians routinely follow up any nonzero response on item 9 of the PHQ-9 with a validated instrument that assesses SI and behavior, such as the Columbia-Suicide Severity Rating Scale [24]. Moreover, the PHQ-9 has not been validated for screening in children younger than 12 years [12]; thus, we recommend the use of measures, such as the Ask Suicide-Screening Questions tool, which has been validated for use in children as young as 8 years old [25]. Third, cross-sectional measurement precludes assessment of persistence or escalation of SI over time or subsequent suicide attempts. Longitudinal studies are needed to characterize the trajectories of SI over time among commercially insured outpatients to identify which subgroups (particularly children, adolescents, and socially vulnerable populations) are at greatest risk of progression from ideation to attempts. Such studies would help clarify the optimal timing and intensity of interventions.

### Conclusions

Despite these limitations, this study provides one of the largest contemporary assessments of SI among commercially insured outpatients in the United States. The findings highlight high and unevenly distributed suicide risk, even among individuals with access to care, and identify clear opportunities for intervention at the clinical, community, and policy levels. Addressing SI earlier, more systematically, and with attention to social and geographic context may be essential to bending the trajectory of suicide rates in the United States.

## Supplementary material

10.2196/101421 Multimedia Appendix 1 Mental health conditions among patients with suicidal ideation by age.

10.2196/101421 Multimedia Appendix 2 Suicidal ideation by social vulnerability.

10.2196/101421 Checklist 1 STROBE checklist.

## Abbreviations

CDC: Centers for Disease Control and Prevention
KFF: Kaiser Family Foundation
PHQ-9: Patient Health Questionnaire-9
SI: suicidal ideation
STROBE: Strengthening the Reporting of Observational Studies in Epidemiology
SVI: Social Vulnerability Index
