# Supplementary material for: Suicidal Ideation Across the Lifespan Among Commercially Insured Outpatients: Retrospective Cohort Study
Source: JMIR Ment Health. 2026 Aug 19;13:e101421. doi: 10.2196/101421 (PMC13489109; doi:10.2196/101421)
Supplement: Supplementary file 1 [file mental-v13-e101421-s003.docx]

**STROBE Statement — Checklist of items that should be included in reports of cross-sectional studies**

*Manuscript: Suicidal Ideation Across the Lifespan: A National Real-World Behavioral Health Cohort (Bravata et al., revised manuscript dated 7/26/2026)*

| **Section/Topic** | **Item No.** | **Recommendation** | **Reported on Page No. / Notes** |
| --- | --- | --- | --- |
| Title and abstract | 1a | (a) Indicate the study’s design with a commonly used term in the title or the abstract | Title (p. 1) and Abstract Methods (p. 3). |
|  | 1b | (b) Provide in the abstract an informative and balanced summary of what was done and what was found | Abstract, pp. 3–4 (structured: Background, Objective, Methods, Results, Conclusions). |
| **Introduction** | | | |
| Background/rationale | 2 | Explain the scientific background and rationale for the investigation being reported | Introduction, pp. 5–6. |
| Objectives | 3 | State specific objectives, including any prespecified hypotheses | Abstract Objective (p. 3); Introduction, final paragraph (p. 6). |
| **Methods** | | | |
| Study design | 4 | Present key elements of study design early in the paper | Methods §2.1 (p. 6). See design-term note at Item 1a. |
| Setting | 5 | Describe the setting, locations, and relevant dates, including periods of recruitment, exposure, follow-up, and data collection | Methods §2.1 (p. 6): national Headway outpatient mental health network; first visits between 1/1/2024 and 10/31/2025. |
| Participants | 6 | (a) Give the eligibility criteria, and the sources and methods of selection of participants | Methods §2.1 (p. 6): first visit with an outpatient mental health provider; commercially insured (Medicaid, Medicare, and self-pay excluded). |
| Variables | 7 | Clearly define all outcomes, exposures, predictors, potential confounders, and effect modifiers. Give diagnostic criteria, if applicable | Methods §2.2–2.3 (pp. 6–7): SI outcome (non-zero PHQ-9 item 9), age, gender, geography, provider type, SVI. |
| Data sources/ measurement | 8 | For each variable of interest, give sources of data and details of methods of assessment (measurement). Describe comparability of assessment methods if there is more than one group | Methods §2.2 (pp. 6–7): Headway digital platform; PHQ-9 item 9 (with scoring); CDC/ATSDR SVI by home zip code; KFF provider-supply data (§2.3, p. 7). |
| Bias | 9 | Describe any efforts to address potential sources of bias | Potential biases (help-seeking sample, single-item measure) are acknowledged in Limitations §4.4 (p. 16). |
| Study size | 10 | Explain how the study size was arrived at | The cohort comprises all eligible patients in the study window (n=189,225; Results §3.1, p. 8). |
| Quantitative variables | 11 | Explain how quantitative variables were handled in the analyses. If applicable, describe which groupings were chosen and why | Age bands and SVI quartiles are evident from Table 1 (pp. 8–10). |
| Statistical methods | 12a | (a) Describe all statistical methods, including those used to control for confounding | Methods §2.3 (p. 7). |
|  | 12b | (b) Describe any methods used to examine subgroups and interactions | Subgroup comparisons by age, gender, geography, SVI, and provider type are presented (Results §3.2, Table 1, Appendices). |
|  | 12c | (c) Explain how missing data were addressed | Available data are reported. Analyses were not adjusted for missing data. |
|  | 12d | (d) If applicable, describe analytical methods taking account of sampling strategy | Not applicable (no complex sampling design). |
|  | 12e | (e) Describe any sensitivity analyses | Not reported. |
| **Results** | | | |
| Participants | 13a | (a) Report numbers of individuals at each stage of study—eg numbers potentially eligible, examined for eligibility, confirmed eligible, included in the study, and analysed | Data are reported for the final analytic cohort (Results §3.1, p. 8). |
|  | 13b | (b) Give reasons for non-participation at each stage | Not reported. |
|  | 13c | (c) Consider use of a flow diagram | Flow diagram not included. |
| Descriptive data | 14a | (a) Give characteristics of study participants (eg demographic, clinical, social) and information on exposures and potential confounders | Results §3.1 (p. 8) and Table 1 (pp. 8–10): age, gender, diagnoses, provider type, SVI. |
|  | 14b | (b) Indicate number of participants with missing data for each variable of interest | Missingness is addressed by reporting by differing denominators (e.g., gender) for the outcomes presented. |
| Outcome data | 15 | Report numbers of outcome events or summary measures | Results §3.2 (pp. 8–10) and Table 1: counts and percentages for each PHQ-9 item-9 response level, overall and by subgroup. |
| Main results | 16a | (a) Give unadjusted estimates and, if applicable, confounder-adjusted estimates and their precision (eg, 95% confidence interval). Make clear which confounders were adjusted for and why they were included | P values are provided (Results §3.2, pp. 8–10); regression analysis are described in §2.3. |
|  | 16b | (b) Report category boundaries when continuous variables were categorized | Table 1 (pp. 8–10): age bands and SVI quartiles are labeled. |
|  | 16c | (c) If relevant, consider translating estimates of relative risk into absolute risk for a meaningful time period | Not applicable; absolute prevalences are reported throughout. |
| Other analyses | 17 | Report other analyses done—eg analyses of subgroups and interactions, and sensitivity analyses | Results §3.2 (pp. 8–10): diagnosis-specific SI by age (Multimedia Appendix 1), SVI subcategory analyses (Multimedia Appendix 2), state-level SI and SI Care Gap Index (Figures 3–4). |
| **Discussion** | | | |
| Key results | 18 | Summarise key results with reference to study objectives | Discussion §4.1 (pp. 11–14). |
| Limitations | 19 | Discuss limitations of the study, taking into account sources of potential bias or imprecision. Discuss both direction and magnitude of any potential bias | Discussion §4.4 (pp. 16–17): generalizability to commercially insured only; single-item SI measure; cross-sectional design. Direction/magnitude of biases (e.g., help-seeking selection, missing gender data) are not quantified. |
| Interpretation | 20 | Give a cautious overall interpretation of results considering objectives, limitations, multiplicity of analyses, results from similar studies, and other relevant evidence | Discussion §4.1–4.3 (pp. 11–16), including comparison with prior work (§4.3). |
| Generalisability | 21 | Discuss the generalisability (external validity) of the study results | Discussion §4.4, first limitation (p. 16). |
| **Other information** | | | |
| Funding | 22 | Give the source of funding and the role of the funders for the present study and, if applicable, for the original study on which the present article is based | Funding section (p. 17): funded by Therapy Match, Inc. (dba Headway); conflicts disclosed (p. 17). |
